# Lempel-Ziv complexity of cortical activity during sleep and waking in rats

**DOI:** 10.1152/jn.00575.2014

**Published:** 2015-02-25

**Authors:** Daniel Abásolo, Samantha Simons, Rita Morgado da Silva, Giulio Tononi, Vladyslav V. Vyazovskiy

**Affiliations:** ^1^Centre for Biomedical Engineering, Department of Mechanical Engineering Sciences, Faculty of Engineering and Physical Sciences (J5), University of Surrey, Guildford, United Kingdom;; ^2^Department of Psychiatry, University of Wisconsin-Madison, Madison, Wisconsin; and; ^3^Department of Physiology, Anatomy and Genetics, University of Oxford, Oxford, United Kingdom

**Keywords:** Lempel-Ziv complexity, local field potentials, neuronal activity, rats, sleep

## Abstract

Understanding the dynamics of brain activity manifested in the EEG, local field potentials (LFP), and neuronal spiking is essential for explaining their underlying mechanisms and physiological significance. Much has been learned about sleep regulation using conventional EEG power spectrum, coherence, and period-amplitude analyses, which focus primarily on frequency and amplitude characteristics of the signals and on their spatio-temporal synchronicity. However, little is known about the effects of ongoing brain state or preceding sleep-wake history on the nonlinear dynamics of brain activity. Recent advances in developing novel mathematical approaches for investigating temporal structure of brain activity based on such measures, as Lempel-Ziv complexity (LZC) can provide insights that go beyond those obtained with conventional techniques of signal analysis. Here, we used extensive data sets obtained in spontaneously awake and sleeping adult male laboratory rats, as well as during and after sleep deprivation, to perform a detailed analysis of cortical LFP and neuronal activity with LZC approach. We found that activated brain states—waking and rapid eye movement (REM) sleep are characterized by higher LZC compared with non-rapid eye movement (NREM) sleep. Notably, LZC values derived from the LFP were especially low during early NREM sleep after sleep deprivation and toward the middle of individual NREM sleep episodes. We conclude that LZC is an important and yet largely unexplored measure with a high potential for investigating neurophysiological mechanisms of brain activity in health and disease.

brain states change continuously on a fast time scale of seconds and minutes, as dictated by inputs from the environment and ongoing behavior. The physiological significance of cortical activity is often unclear, but it has been associated with ongoing sensory input or offline information processing (Dang-Vu et al. 2008; Harris and Mrsic-Flogel 2013; Hasenstaub et al. 2007; Kudrimoti et al. 1999; Luczak et al. 2013). On a slower time scale, such changes are shaped by regular transitions between waking and sleep, which are governed by the circadian clock, time of day, and preceding sleep-wake history (Brown et al. 2012; Fisher et al. 2013). One of the most pronounced temporal variations in sleep process refers to so-called homeostatic regulation of sleep, which has been documented in several mammalian and nonmammalian species (Cirelli and Tononi 2008; Jones et al. 2008; Tobler 2005; Vyazovskiy and Harris 2013). It is manifested in increased sleep “intensity” after prolonged waking, measured as spectral electroencephalogram (EEG) power in slow (<4 Hz) frequency range, so-called slow-wave activity (SWA) (Achermann et al. 1993; Deboer 2013; Porkka-Heiskanen et al. 2013). The homeostatic changes in spectral EEG power are accompanied by changes in the amplitude and frequency of individual slow-waves and in their spatial dynamics (Nir et al. 2011; Riedner et al. 2007; Vyazovskiy et al. 2011b). However, the relevance of such changes for sleep regulatory mechanisms and sleep function remains poorly understood. It has been suggested that homeostatic sleep regulation reflects synaptic plasticity (Tononi and Cirelli 2014; 2006) and prophylactic cellular maintenance (Vyazovskiy and Harris 2013), or that it is relevant for memory consolidation (Rasch and Born 2013).

Notably, the signals recorded from the neocortex in different vigilance states—waking, non-rapid-eye movement (NREM) and rapid eye movement (REM) sleep are markedly different in terms of the total signal amplitude and frequency content (Vyazovskiy et al. 2009b). Developing new metrics that are based on parameters other than amplitude and frequency is essential, as it will open new opportunities for understanding the functional significance of brain activity in health and disease. For example, this is important when the absolute amplitude and/or spectral power of the EEG signal in specific frequency bands is different between conditions or population groups, as is apparent when the comparison spans age (Carrier et al. 2011; Huber and Born 2014), sex differences, or ethnic differences (Carrier et al. 2001; Latta et al. 2005; Mokhlesi et al. 2012) or concerns pharmacological treatments (Dijk et al. 2010) or sleep disorders (Krystal et al. 2002; Spiegelhalder et al. 2012).

More generally, the regulatory mechanisms of sleep remain, in many parts, poorly understood because of an enormous neuroanatomical complexity of circuits relevant for sleep regulation and the multitude of spatial and temporal scales at which sleep regulation is manifested (Brown et al. 2012; Olbrich et al. 2011; Vyazovskiy and Delogu 2014). Therefore, the development of novel signal analysis approaches will not merely provide additional information but may appear crucially important for understanding the general principles underlying sleep dynamics.

Nonlinear analysis of EEG or local field potential (LFP) signals with Lempel-Ziv complexity (LZC) (Lempel and Ziv 1976) appears to provide valuable novel insights (Abásolo et al. 2006; Arnold et al. 2012; Li et al. 2008; Radhakrishnan and Gangadhar 1998; Zhang et al. 2001), complementary to those obtained with spectral analyses. Several complexity measures are available (Tononi et al. 1998), such as dimensional complexity, corresponding to the well-known nonlinear method of correlation dimension, Kolmogorov or algorithmic complexity, reflecting the shortest computer program that can generate a binary sequence, and neural complexity, defined in terms of integration or in terms of mutual information. LZC is a nonparametric measure of complexity for finite sequences in Kolmogorov's sense, and is related to the number of distinct substrings or patterns within the sequence and the rate of their occurrence along the sequence (Lempel and Ziv 1976). In LZC, the original signal is first converted into a binary sequence by using a process called coarse-graining, and then the number of different substrings or patterns in the binary sequence is computed. More complex signals would have more different patterns than simpler, more regular signals.

The application of nonlinear time series analysis metrics to physiological signals is a valuable tool because “hidden information” related to underlying mechanisms can be obtained (Pincus and Goldberger 1994). For example, there is recent evidence that LZC can highlight state-dependent changes in information content in spike trains recorded in the primary visual cortex in chronically implanted rats (Arnold et al. 2012). Specifically, an increase about 30% in LZC was found at the transition from sleep to waking, while going back to sleep was associated with a comparable decrease. In addition, an LZC-based index called the perturbational complexity index was proposed and validated as a measure of consciousness in an extensive set of data obtained in patients recorded under anesthesia, in coma, persistent vegetative state, and during sleep (Casali et al. 2013).

While LZC has appeared a very promising tool for understanding neural dynamics in vivo in awake animals (Amigo et al. 2004; Arnold et al. 2012; Szczepanski et al. 2003), more research is necessary to reach a better understanding of the validity and relevance of nonlinear metrics for analyzing electrical brain activity during sleep. It should be kept in mind that the choice of coarse-graining method in the calculation of LZC may have an influence on the results and their interpretability. Although different methods of coarse-graining have been performed on the electrocardiogram of human patients (Zhou et al. 2011), such analyses have not been previously applied to cortical LFPs and neuronal spike trains in spontaneously sleeping and awake rats. In addition, LZC approach has not been applied previously to investigate the effects of preceding sleep-wake history on LFPs and neuronal activity.

## MATERIALS AND METHODS

### 

#### Animals.

Adult male Wistar-Kyoto (WKY) rats were used for this study (*n* = 11 in total). All rats were housed individually in transparent Plexiglas cages. Lighting and temperature were kept constant (12:12-h light-dark cycle, with light on at 10 AM, 23 ± 1°C), and food and water were available ad libitum and replaced daily at 10 AM.

#### Surgical procedures.

All procedures related to animal handling, recording, and surgery followed the National Institutes of Health's “Guide for the Care and Use of Laboratory Animals” and were approved by the Institutional Animal Care and Use Committee (IACUC). One day before surgery animals received an intraperitoneal dose of dexamethasone (0.2 mg/kg ip) to suppress local immunological response (Spataro et al. 2005; Zhong and Bellamkonda 2007). Under deep isoflurane anesthesia (1.5–2% volume), polyimide-insulated tungsten microwire arrays were implanted in the frontal cortex (B: +1–2 mm, L: 2–3 mm). The arrays were 16-channel (2 rows each of 8 wires) polyimide-insulated tungsten microwire arrays [Tucker-Davis Technologies (TDT), Alachua, FL; wire diameter, 33 μm; electrode spacing, 175–250 μm; row separation, L-R: 375–500 μm; D-V: 0.5 mm], according to the surgical implantation guidelines (Neuronexus Technologies, Ann Arbor, MI) (Kralik et al. 2001). Dexamethasone (0.2 mg/kg) was given with food pellets every day for the duration of the experiment. The surgical procedure was performed in sterile conditions, using ethylene oxide-sterilized materials. An ∼2 × 2 mm craniotomy was made using first a 1.4-mm drill bit and then a 0.5-mm drill bit, with the aid of a high-speed surgical drill. The hole was adjusted to the size of the array by removing the remaining fragments of the bone, and the dura was dissected. The electrode array was advanced into the brain tissue by penetrating the pia mater, making an effort to avoid vascular damage (Bjornsson et al. 2006). Electrode insertion was achieved by advancing the electrode array until both rows of the arrays were at the level of deep cortical layers (∼1.5 mm below the pial surface). The final position of the array was adjusted by withdrawing or lowering it slowly (∼50-μm steps) until most channels showed robust single-unit or multiunit activity. The final position of the wires was identified to reside in deep layers, as judged from the positivity of LFP slow waves during NREM sleep, corresponding to the neuronal population silent periods. At this stage, special care was taken to avoid displacing the array in the horizontal dimension. The two-component silicon gel (KwikSil; World Precision Instruments, Sarasota, FL) was used to seal the craniotomy and protect the surface of the brain from dental acrylic. After ∼10 min, required for the gel to polymerize, dental cement was gently placed around the electrode, fixing the array to the skull. The ground and reference screw electrodes were placed above the cerebellum, and additional anchor screws were placed in the frontal bone.

#### Experimental design.

About 1 wk was allowed for recovery after surgery, and experiments were started only after the sleep/wake cycle had fully normalized, as evidenced by the entrainment of sleep and wake by the light-dark cycle and the homeostatic time course of cortical LFP SWA (0.5–4 Hz). After a stable baseline, animals were recorded during 4 h of prolonged waking, followed by an undisturbed recovery period (Vyazovskiy et al. 2011b). Prolonged waking began at light onset and involved continuous observation of the animal and its polysomnographic recording. The animals were given a novel object to play with, or were activated by acoustic stimuli (e.g., tapping on the cage) whenever they assumed a sleep posture, or started exhibiting electrographic signs of drowsiness (LFP slow waves or low-tone EMG). Rats were not touched or handled directly. Objects included paper tissue and paper towels, bedding material transferred from other cages, and toys of various shapes and sizes.

#### Signal processing and analysis.

Data acquisition and online spike sorting were performed with the Multichannel Neurophysiology Recording and Stimulation System (TDT). Spike data were collected continuously (sampling frequency: 25 kHz, bandwidth: 300 Hz–5 kHz), concomitantly with the LFPs from the same electrodes (sampling frequency: 256 Hz, bandwidth: 0.1–100 Hz) and the EMG (sampling frequency: 256 Hz, bandwidth: 10–100 Hz). The online spike sorting was performed with OpenEx software (TDT), by applying a voltage window through which the signal must pass. Amplitude thresholds for online spike detection were set manually and allowed only crossings of spikes with peak amplitude exceeding the amplitude of noise by at least a factor of 2. Such thresholding allowed excluding the low-amplitude noise and most of the high-amplitude artifacts related to chewing and grooming. Since extracellular multiunit activity (MUA) signals are usually asymmetric, the detection threshold (chosen individually in the range of 20–30 μV) was applied to the side where spike waveforms exhibited greater deflection (Rasch et al. 2008). Whenever the recorded voltage exceeded a predefined threshold, a segment of 46 samples (0.48 ms before and 1.36 ms after the threshold crossing) was extracted and stored for later use together with the corresponding time stamps. Spike data were then subjected to an offline sorting procedure (Vyazovskiy et al. 2011b). All individual neurons were carefully screened to avoid contamination of the sorted units, especially of small amplitudes, by MUA: units showing relatively high proportion (>1–2%) of short refractory periods (<2.5 ms) and no major peaks on the distribution of interspike intervals were discarded from the analysis of firing rates (Vyazovskiy et al. 2009b). The LFP power spectra were computed by a fast Fourier transform routine for 4-s epochs (Hanning window, 0.25-Hz resolution). On the basis of the spectra of wake and sleep LFP during sleep deprivation and recovery, two frequency bands were selected for the analyses: high delta/low theta band (2–6 Hz) in waking, and SWA (0.5–4.0 Hz) in NREM sleep (Vyazovskiy et al. 2011b). To emphasize the overall magnitude of change, for some of the analyses, relative spectral values were calculated by normalizing them within an individual as the percentage of the mean value of the power in the corresponding frequency band over all the recording periods.

#### Scoring vigilance states and behavioral analysis.

Prior to signal analysis, vigilance states were identified for consecutive 4-s epochs. To do so, signals were loaded with custom-written MatLab programs using standard TDT routines, and subsequently transformed into the European data format using Neurotraces software (www.neurotraces.com). Sleep stages were scored off-line by visual inspection of 4-s epochs (SleepSign, Kissei), where the LFP, electromyogram (EMG), and spike-activity were displayed simultaneously. Waking was characterized by low-voltage, high-frequency LFP patterns and phasic EMG activity. Epochs of eating, drinking, and intense grooming (< 5%) were carefully excluded, since during those periods, MUA is contaminated by movement artifacts, for example, due to chewing, precluding reliable isolation of individual spikes. NREM sleep was characterized by the occurrence of high-amplitude LFP slow waves and low tonic EMG activity (Leemburg et al. 2010; Vyazovskiy et al. 2009b). During REM sleep, the LFP was similar to that during waking, but only heart beats and occasional twitches were evident in the EMG signal.

Two data sets were analyzed. One data set consisted of selected consolidated artifact-free episodes of waking, NREM, and REM sleep, recorded during an 8-h period after 4-h sleep deprivation. The total amount of vigilance states in this data set contributing to the analysis was 476.57 ± 67.5, 709.71 ± 158.4, and 194.67 ± 19.5 s for waking (W), NREM sleep (N), and REM sleep (R), respectively (*n* = 11 rats). In a subset of animals, long-term daily changes in LZC were calculated, for which continuous 12-h recordings obtained during an undisturbed baseline day (*n* = 3 rats, wake: 3.9 ± 0.5 h, NREM sleep: 6.8 ± 0.4 h, REM sleep: 1.3 ± 0.2 h) or 4-h sleep deprivation + 8 h of undisturbed recovery (*n* = 5 rats, wake: 6.1 ± 0.1 h, NREM sleep: 4.8 ± 0.1 h, REM sleep: 1.0 ± 0.1 h) were included.

#### Spike sorting.

Spikes were first detected using a threshold-based technique previously described (Vyazovskiy et al. 2009b). Only those spikes for which waveform shape was consistent with typical recordings and the signal-to-noise ratio was greater than 2 were considered. Principal components (PCs) were extracted (Lewicki 1998), and clustering was performed on the basis of the split-and-merge expectation-maximization (SMEM) algorithm (Tolias et al. 2007; Ueda et al. 2000). This algorithm operates on Gaussian mixtures by iteratively splitting and merging Gaussian clusters, until convergence of a maximization index is reached (only the split phase was employed to reduce computational time). Although the merge step should help avoiding convergence to local maxima (Ueda et al. 2000), we empirically verified that, on our data, the split step alone was sufficient to correctly estimate the number of clusters. To obtain a satisfactory clustering quality, parameters were initialized as follows: *1*) the number of PCs was set to 3, accounting on average for ∼70% of the total variance; *2*) the threshold for the algorithm convergence was set to 0.01 (this value influences the number of detected clusters); and *3*) the threshold for classification was set to 0.1, i.e., all spikes with a probability lower than 10% of belonging to a cluster were discarded. All clusters were checked post hoc, and clusters with standard deviation greater than 20% of mean spike amplitude were rejected.

#### Lempel-Ziv complexity.

Lempel-Ziv complexity (LZC) was computed for the LFPs and spike trains. LZC is a method of symbolic sequence analysis that measures the complexity of finite length sequences (Lempel and Ziv 1976) by computing the number of distinct substrings and the rate of their recurrence along the given sequence (Radhakrishnan and Gangadhar 1998). With LFPs, the signal has to be first converted into a sequence with a finite number of symbols by a process called coarse-graining of the signal. In this study, a binary conversion was used and two different coarse-graining options—the median, proven to be robust to outliers, or k-means as the threshold in the symbolization of the original signal (Zhou et al. 2011)—were used to create the symbolic sequence from each 4-s epoch.

#### Median.

The signal is converted into a binary sequence *P* = *s*(1), *s*(2), . . ., *s*(*n*) by comparing each sample of the signal, *x*(*i*), with a the median of the time series *T*_*d*_, with *s*(*i*) then given by (Zhang et al. 2001):

(1) $$s\left(i\right)=\{\begin{array}{cc} 0 & if\;x\left(i\right)<T_{d}\\ 1 & if\;x\left(i\right)\geq T_{d} \end{array}$$

#### k-means.

This approach is based on the grouping of data around centroids corresponding to points around which most of the data is agglomerated (Zhou et al. 2011). For binary sequences, the number of centroids is 2, and they can be set in the initial iteration of this coarse-graining method as:

(2) $$z_{1}\left(1\right)=x_{m}+\varepsilon \cdot x_{m}$$

(3) $$z_{2}\left(1\right)=x_{m}-\varepsilon \cdot x_{m}$$

where *ε* = 0.005, *x*_*m*_ is the mean of the data points from the original signal, *x*(*i*) (Zhou et al. 2011), and *z*_1_ and *z*_2_ are the initial positions of the two centroids. Distance from each data point *i* to centroids *z*_1_ and *z*_2_ (*D*_1_ and *D*_2_, respectively) are then calculated as:

(4) $$\begin{array}{c} D_{1}^{i}={\left\|x\left(i\right)-z_{1}\left(1\right)\right\|}^{2}\\ D_{2}^{i}={\left\|x\left(i\right)-z_{2}\left(1\right)\right\|}^{2} \end{array}$$

The signal is converted into a binary sequence *P* = *s*(1), *s*(2), . . ., *s*(*n*) following a minimum distance criterion, with *s*(*i*) given by (Zhou et al. 2011):

(5) $$s(i)=\{\begin{array}{cc} 1 & if\;D_{1}^{i}<D_{2}^{i}\\ 0 & if\;D_{1}^{i}\geq D_{2}^{i} \end{array}$$

Group 1 contains all samples assigned with symbol 1, while group 2 refers to samples assigned with a symbol 0 after this initial iteration. In a new iteration, two new centroids have to be defined. For each group, the new centroid is the average coordinate among all of the members in the group. *Equations 4* and *5* are then reapplied to find the new distance values and the new symbolic sequence *P*, respectively. The procedure has to be repeated until *z*_1_(*j*+1) = *z*_2_(*j*) for all *j*.

LZC of these binary sequences obtained with k-means (LZCkm) and median (LZCm) was computed using the parsing process suggested by Lempel and Ziv (1976), where the binary sequence *P* is scanned from left to right, and a complexity counter *c*(*n*) is increased every time a new subsequence of consecutive characters is found. To have a LZC independent of the signal length, LZC was normalized using the upper bound of the complexity, generally given by (Lempel and Ziv 1976):

(6) $$b\left(n\right)\equiv \frac{n}{\log_{\alpha }\left(n\right)}$$

where *α* is the number of symbols in the alphabet (*α* = 2 for the binary conversion considered here). The normalized LZC can be defined as (Zhang et al. 2001):

(7) $$C\left(n\right)=\frac{c\left(n\right)}{b\left(n\right)}$$

**Fig. 1.**
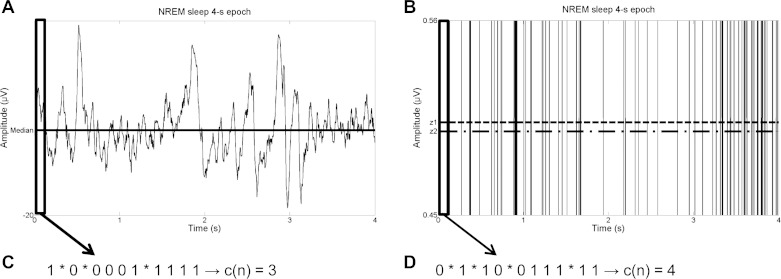
*A*: representative 4-s local field potential (LFP) epoch recorded from the frontal cortex in a freely behaving rat in non-rapid eye movement (NREM) sleep with the median used in the coarse-graining of the signal. *B*: same epoch showing the initial centroids *z*_1_ and *z*_2_ for the k-means coarse-graining of the signal (zooming in the *y*-axis for visualization purposes). *C*: first values of the binary sequence obtained with the median as the threshold from the highlighted window of data from the epoch, showing the different subsequences detected by the Lempel and Ziv algorithm separated with asterisks. *D*: first values of the binary sequence obtained with k-means from the highlighted window of data from the epoch, showing the different subsequences detected by the Lempel and Ziv algorithm separated with asterisks. Note that for *C* and *D*, the last characters (1111 and 11, respectively) are not new subsequences.

Figure 1 illustrates how LZC can be computed for a representative 4-s LFP epoch in NREM sleep. Spike trains, on the other hand, were already binary sequences (at 500-Hz resolution, for each 2-ms window, there was 0 if no spikes happened or 1 if a spike occurred). Therefore, the initial coarse-graining for the symbolization of the sequence was not needed, and the Lempel-Ziv algorithm from 1976 was directly applied to the spike trains sequence to estimate its complexity. As for LFP SWA, some of the analyses have been performed on relative values of LZC, which were calculated by normalizing them within an individual for each time interval, as the percentage of the mean value over the entire recording period.

## RESULTS

### 

#### State-dependent changes in LZC of the cortical signals.

**Fig. 2.**
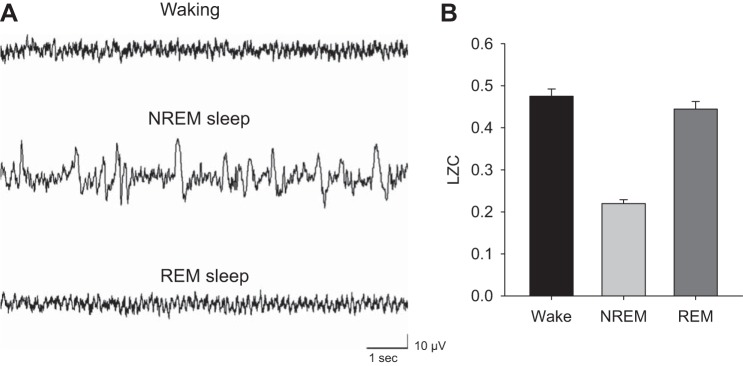
*A*: representative 8-s local field potential (LFP) traces recorded from the frontal cortex in a freely behaving rat in spontaneous waking, NREM, and rapid eye movement (REM) sleep. *B*: average Lempel-Ziv complexity (LZC) values, computed by a k-means coarse-graining technique, in the three behavioral states (*n* = 11 rats; values are expressed as means ± SE).

First, LZC values computed for the LFPs of consecutive 4-s epochs in waking, NREM sleep, and REM sleep were compared between vigilance states, using the first data set (see materials and methods). The three vigilance states are distinguished by specific changes both in the total amplitude of cortical signals and by their frequency composition (Fig. 2*A*). It was found that average LZC values, computed over consecutive 4-s epochs using k-means coarse-graining approach, were substantially lower in NREM sleep compared with waking and REM sleep (Fig. 2*B*). The two different coarse-graining methods were then used to compare if vigilance state-specific differences are affected by the algorithm. This appeared not to be the case, as the mean values were virtually identical between k-means (LZCkm) and median (LZCm) coarse-graining approaches (not shown).

**Fig. 3.**
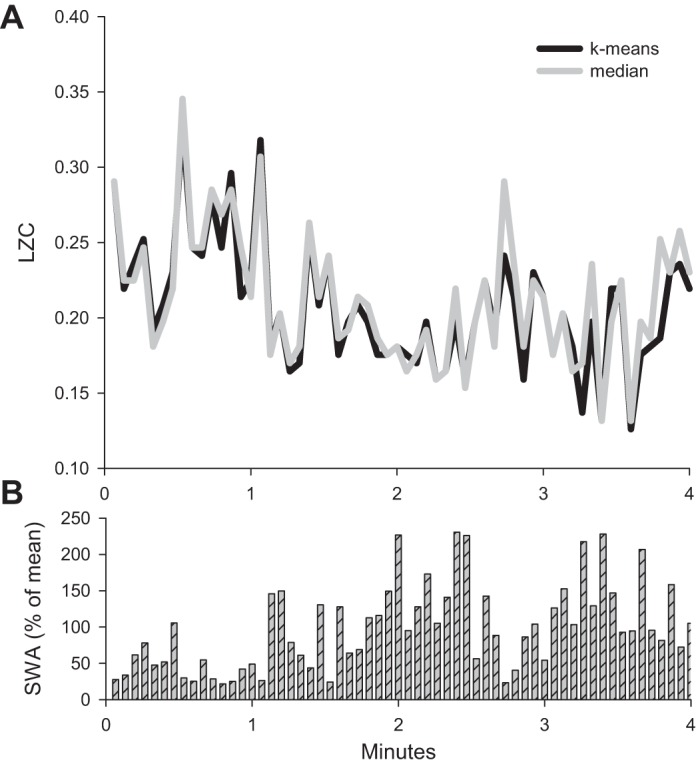
*A*: LZC values computed with different coarse-graining approaches shown for consecutive 4-s epochs during one representative NREM sleep episode. Note that occasionally the values of LZC computed with k-means approach deviate from corresponding values obtained with median technique. *B*: corresponding values of LFP slow-wave activity during the same NREM sleep episode. The values of sleep wave activity (SWA) are expressed as % of mean SWA value over the entire 4-min episode.

Although, on average, LZCkm and LZCm values were similar, during a subset of 4-s epochs, the values deviated, and this was especially apparent in NREM sleep (Fig. 3). On average, the proportion of epochs, for which the absolute difference (either positive or negative) between LZCkm and LZCm values exceeded 10% was 2.7 ± 2.4%, 16.8 ± 4.3%, and 1.3 ± 1.2% of all 4-s epochs in waking, NREM sleep, and REM sleep, respectively. Interestingly, in NREM sleep, the epochs with substantial differences between the LZCkm and LZCm values also showed systematic differences in terms of signal variance and LFP power in the slow-wave (SWA: 0.5–4 Hz) range (Fig. 3). Specifically, both signal variance and SWA were higher during those epochs in which LZCkm and LZCm were different by at least 10% [LFP variance: 81.8 ± 16.9 vs. 58.3 ± 13.9 μV, *P* = 0.0351, paired *t*-test; relative SWA: 121.5 ± 2.6 vs. 95.1 ± 1.5 (% of mean SWA over all epochs), *P* = 7.3030e-004, paired *t*-test]. Thus, while k-means and median coarse-graining approaches yield, on average, similar results, caution is warranted in choosing one method over the other, as it may lead to overestimation or underestimation of the complexity depending on a vigilance state and spectral content of the signals.

**Fig. 4.**
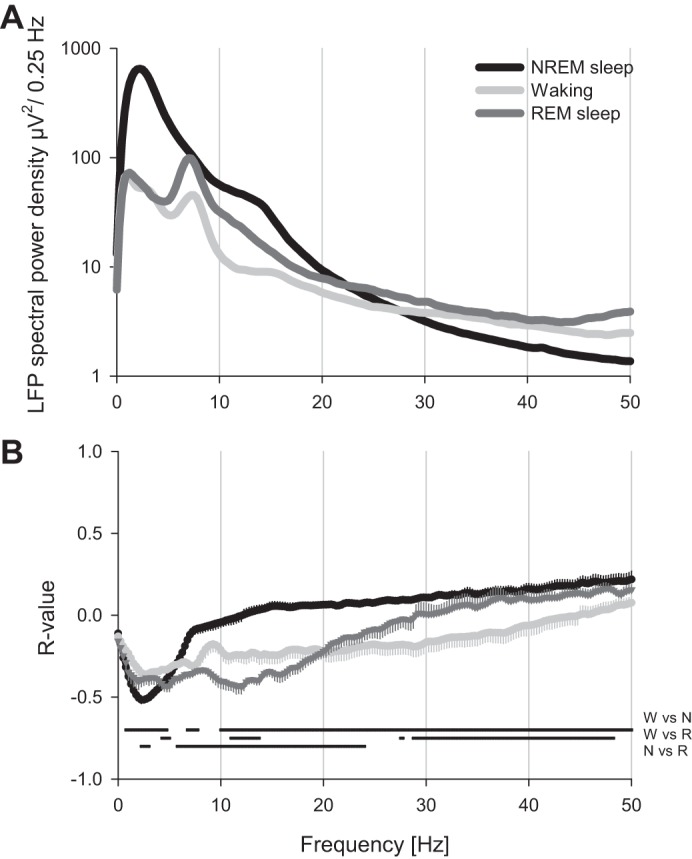
*A*: LFP spectral power in NREM sleep, waking and REM sleep during 12-h undisturbed baseline recording in one representative rat. *B*: *R* values of Pearson product-moment correlation between LZC values (computed with k-means approach) and corresponding power spectral values over *n* = 5 rats (mean values ± SE). Note that there is a strong negative correlation between LFP power in slow-frequency range and LZC values, especially apparent in NREM sleep.

The values of LZC were far from being stable within a state, but varied substantially. The magnitude of change within a state was 24.2 ± 2.04% in NREM sleep, and reached 8.4 ± 0.6 and 10.6 ± 0.8% in waking and REM sleep, respectively. Similarly, in another study in which LZC was estimated from neuronal spike trains, large within-state fluctuations were also apparent (Arnold et al. 2012). This was consistent with the changes in spectral characteristics of brain signals, which vary continuously within a state (e.g., Fig. 3*B*). To systematically assess this relationship, we computed LZC values and power spectra over the baseline 12-h period in a subset of animals. In all animals, the LZCkm values in NREM sleep correlated strongly and negatively with LFP SWA, while a weak positive correlation was apparent at faster frequencies (Fig. 4). Notably, the negative correlation between LZCkm values and slow LFP frequencies was also apparent in REM sleep, but not systematically observed in waking.

As absolute spectral EEG values often show high interindividual variability, various normalization procedures are employed. These are often suboptimal, as they may eliminate potentially physiologically relevant differences. We hypothesized that LZC, which is relatively independent from the absolute amplitude values, is a potentially useful approach to address shortcomings of conventional normalization techniques. To investigate this aspect, we compared the variability between individual animals in terms of absolute spectral EEG power and LZC values. Specifically, we focused on the sleep deprivation period and found that wake EEG power in the slow theta-range (2–6 Hz) showed much higher interindividual variability compared with LZC values. Expressed as a percentage of the standard deviation and the mean, the variability was only 5.57% for LZC, while it reached 29.3% for the EEG power.

#### Homeostatic sleep pressure is reflected in LZC of brain signals.

**Fig. 5.**
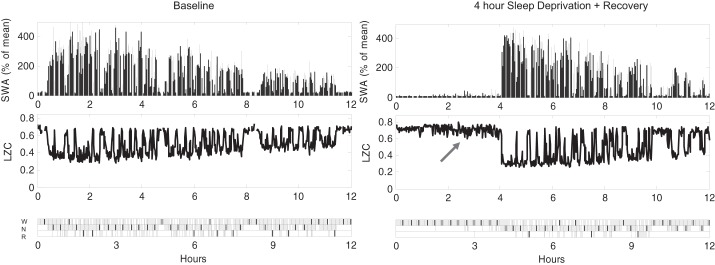
Time course of LFP slow-wave activity (spectral power between 0.5 and 4 Hz) during undisturbed 12-h baseline sleep period and during 4-h sleep deprivation (SD) followed by 8 h of recovery sleep in one individual rat. SWA for each 1-min epoch is expressed as a percentage of the mean value over the entire recording period. Note that SWA is invariably high at the beginning of spontaneous sleep and after sleep deprivation, and it shows a progressive decline during sleep. LZC values (k-means approach) show a decline during sleep, which is especially pronounced in early sleep after sleep deprivation. It is also apparent that LZC values become progressively more variable in the course of sleep deprivation (arrow), possibly indicating state instability.

**Fig. 6.**
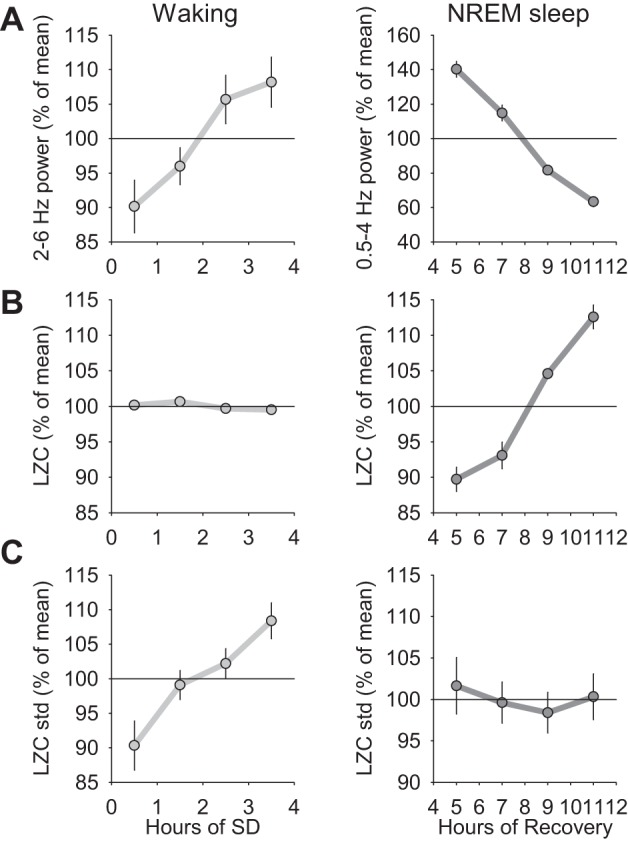
*A*: time course of spectral EEG power between 2 and 6 Hz (waking) and 0.5 and 4 Hz (NREM sleep) during 4 h of sleep deprivation and 8 h of recovery, respectively. Mean values (*n* = 5 rats). *B*: corresponding values of mean LZC, calculated with a k-means coarse-graining approach. *C*: corresponding values of SD of LZC across all of the epochs for each time interval. To emphasize the overall magnitude of change, all variables are normalized within an individual as % of their mean value over the entire recording period, prior to averaging between animals.

One of the conspicuous characteristics of NREM sleep EEG or LFP under physiologically elevated sleep pressure is increased SWA, which is accounted for by higher amplitude and more frequent slow waves (Riedner et al. 2007; Vyazovskiy et al. 2007). Figure 5 illustrates a representative 12-h profile of cortical LFP slow-wave activity and the corresponding LZCkm values during an undisturbed 12-h baseline period and after sleep deprivation. We found that early NREM sleep with high SWA was characterized by substantially lower values of LZCkm, which increased progressively in the course of recovery sleep (Fig. 6). Therefore, we hypothesized that LZC is a sensitive metric for preceding sleep-wake history, which is relatively independent of the absolute levels of SWA. To address this hypothesis, we matched individual 4-s epochs in early (first 2 h after sleep deprivation) and late sleep (last 2 h of the light period) by the values of SWA and compared the corresponding LZCkm values. Intriguingly, even when averaged SWA values were virtually identical, LZCkm values were still significantly lower by 12.3 ± 1.9% in the initial recovery sleep (*P* = 0.017; paired *t*-test). Moreover, when only epochs with high SWA (more than mean + 1 SD) were included in the calculation, the difference appeared even more pronounced (−19.6 ± 2.0%, *P* = 0.0001).

It is well known that the EEG or LFP amplitude is substantially lower in waking compared with NREM sleep (e.g., Figs. 4 and 5). Therefore, absolute changes in spectral characteristics of the brain signal during waking are often considered negligible, as they are much smaller than the respective changes in NREM sleep. Using metrics that are relatively independent of the absolute amplitude of the signal becomes especially valuable to obtain additional insights. When we computed the time course of slow (2–6 Hz) frequency LFP power within the 4 h of sleep deprivation, as expected, we found it to increase progressively with time awake (Fig. 6*A*; ANOVA for repeated measures, factor “time”; *P* = 0.02). However, the values of LZCkm showed no systematic change resulting in a virtually flat line (Fig. 6*B*; *P* = 0.53). In contrast, the epoch-to-epoch variability, computed as a standard deviation of LZCkm values within each hourly interval, showed a pronounced increment across the period of sleep deprivation, with a magnitude compared with the change in slow LFP power (Fig. 6*C*; *P* = 0.01).

The results obtained in waking during sleep deprivation showed interesting differences from the changes observed in subsequent recovery NREM sleep. First, as expected, the initial values of SWA in recovery NREM sleep were high and showed a progressive decline reaching values ∼2 times lower by the end of the light period (ANOVA for repeated measures, factor “time”; *P* < 0.001). At the same time, average LZCkm values showed opposite changes starting from low values, which increased on average slightly more than by 20% within the 8-h recovery period (Fig. 6*B*, *P* < 0.001). However, this was not reflected in the variability between individual epochs within the time intervals (Fig. 6*C*; *P* = 0.9).

#### Intraepisodic dynamics of signal complexity.

Having analyzed the global state-dependent changes in the information content of the LFP signals and its slow homeostatic changes across the day, the changes in LZC on a finer time scale were investigated next within individual sleep episodes. Understanding the mechanisms underlying such changes is important, as the processes of falling asleep and transitioning between brain states are usually gradual, rather than abrupt, and both cortical and subcortical mechanisms may be involved (Fort et al. 2009; Schwierin et al. 1999; Vyazovskiy et al. 2014).

**Fig. 7.**
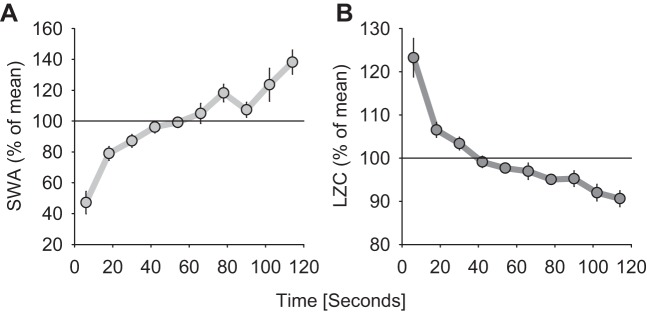
*A*: time course of spectral EEG power between 0.5 and 4 Hz during the first 2 min after NREM sleep episode onset during recovery after sleep deprivation. Mean values (*n* = 5 rats, all NREM sleep episodes >2 min are included). *B*: corresponding values of mean LZC (k-means coarse-graining approach). Note that while SWA increases progressively within an episode, the information content shows a decrease.

First, the dynamics of LZC within individual NREM sleep episodes was investigated. As is well documented, spectral power in slow-wave range shows systematic changes within a NREM sleep episode (Vyazovskiy et al. 2004; Vyazovskiy et al. 2009a). As expected, in the present data set, it was apparent that SWA starts initially at low levels and shows a gradual buildup, until reaching a plateau (Fig. 7*A*, ANOVA for repeated measures, factor “time”, *P* < 0.001). The time course of LZCkm after the NREM sleep episode onset was a mirror image of the change in SWA, being high initially and declining progressively within the first 1–2 min of the NREM sleep episode (Fig. 7*B*; *P* < 0.001). The relative change in SWA was substantial—the magnitude of the change within a NREM sleep episode was about two-fold (*P* = 1.0366e-009). In contrast, the decline of LZCkm was modest, showing a decrease by no more than ∼20%, albeit the change was also highly significant statistically (*P* = 4.6442e-011).

#### LZC in neuronal spike trains.

**Fig. 8.**
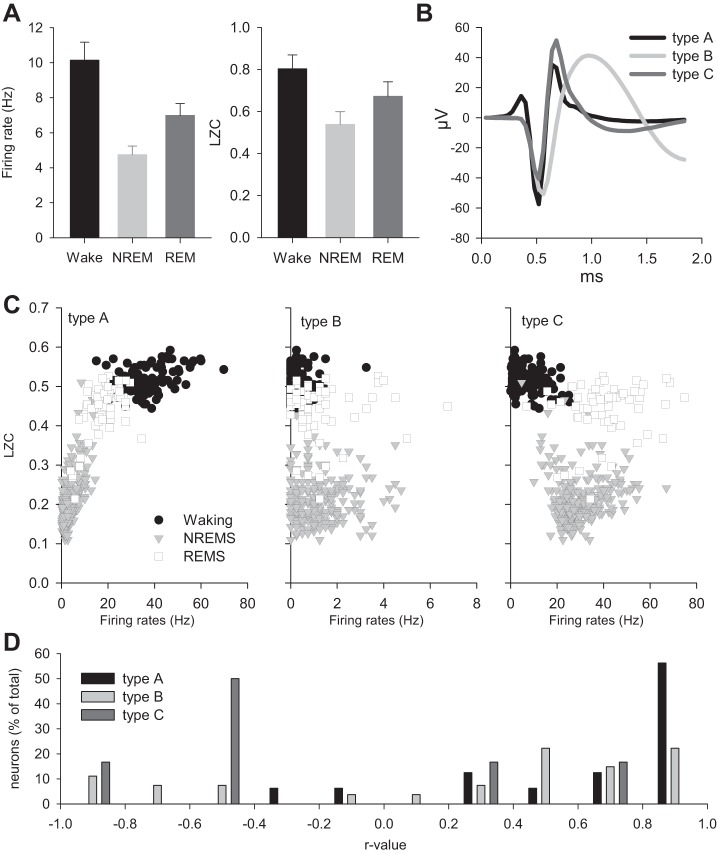
*A, left*: mean neuronal firing rates in waking, NREM, and REM sleep. Mean values ± SE (*n* = 11 rats). *Right*: corresponding LZC computed from spike trains based on 4-s epochs (k-means approach). *B*: typical representative waveforms of three types of neurons defined on the basis of the shape of extracellular action potentials. *C*: scatterplots of average LZCkm calculated for 4-s epochs are plotted against firing rates of three individual neurons belonging to the types shown in *B*. Epochs in waking, NREM, and REM sleep are plotted separately to reveal vigilance state-specific differences in both the firing rates and the LZC. *D*: distribution of individual neurons as a function of the *r* value of the correlation between their firing rates and LZC (50 well-isolated neurons in total over *n* = 6 rats). Note that firing activity of most neurons of type A correlates positively with LZC calculated over corresponding spike trains, while neurons of type B and C show a less clear relationship.

Finally, we investigated whether the changes in the LZC derived from the LFP parallel to those obtained on the basis of the dynamics of multineuron activity. As has been shown before, the average firing rates in “active” brain states, such as waking and REM sleep, are usually higher than the corresponding values in NREM sleep (Vyazovskiy et al. 2009b). This is likely to be explained by the regular occurrence of neuronal OFF periods, when the local or global population quasi-synchronously enters a down-state, when no spiking activity is occurring (Vyazovskiy et al. 2009b). Average neuronal firing rates were consistently highest in waking, and lowest in NREM sleep with the values of REM sleep usually intermediate or closer to those observed in waking. Computing the corresponding LZC values revealed that complexity of the signal was lowest in NREM sleep, somewhat higher in REM sleep, and highest in wakefulness (Fig. 8*A*). Thus, LZC values correlate negatively with LFP power in slow-wave range (Fig. 4) and positively with neuronal firing rates (Fig. 8).

To investigate whether there is an unspecific correlation between neuronal activity and LZC, in a subset of animals (*n* = 6), we selected one episode of each vigilance state (waking, NREM sleep, and REM sleep), and calculated neuronal firing rates for each individual well-isolated neuron (50 in total) and plotted them against values of LZC calculated from a spike train of the corresponding 4-s epoch. As previously reported (Vyazovskiy et al. 2013), three types of neurons have been identified on the basis of the extracellular action potential spike wave form. Many of those neurons, which had narrow spike and rapidly decaying afterhyperpolarization (type A) showed a strong state dependency and a positive correlation with LZC. In contrast, the other two types (B and C), showed a much weaker relationship and only a fraction of those correlated positively or negatively. This result suggests that, although, on average, state dependency of neuronal firing rates look similar to LZC calculated from spike trains, individual neurons often deviate substantially.

Finally, we addressed whether different coarse-graining approaches that are applied to LFPs are associated with differences in concomitant neuronal activity. We found that in NREM sleep, the average absolute firing rates were significantly lower during epochs when the difference between LZCkm and LZCm values of LFPs exceeded 10%, compared with those epochs when LZCkm and LZCm values of LFPs were similar (5.9 ± 0.7 Hz vs. 6.54 ± 0.7 Hz; *P* = 0.0461, paired *t*-test). This result is consistent with higher SWA during those epochs (Fig. 3) and suggests that the choice of the coarse-graining technique may yield different results depending on the structure of the signal under scrutiny.

## DISCUSSION

In this study, a detailed analysis of LZC derived from cortical LFPs neuronal spike trains recorded from the frontal primary motor cortex in freely behaving WKY rats was performed. Results corroborate recent findings by Arnold et al. that information content in spike trains recorded from the primary visual cortex in adult male Lister hooded rats (Arnold et al. 2012) increases during waking and decreases during slow wave sleep by about 30%. Notably, no visual task was used in that study, and the experiments were performed in the dark, in the absence of any visual stimulation, suggesting that the changes are not driven by processing of external information but rather arise from intrinsic state-dependent mechanisms. Likewise, in our study, in which signals from the primary motor cortex were recorded, a substantial change was found between NREM and REM sleep, despite the absence of overt motor activity. Moreover, we found that early NREM sleep after sleep deprivation is characterized by decreased LZC, which correlated inversely with the well-established marker of sleep homeostasis—LFP spectral power in slow-wave range. We found that different approaches of coarse-graining have only marginal overall influence on the resulting values of LZC. However, in those cases in which there was an effect of coarse-graining technique, especially during NREM sleep, signals also differed in terms of temporal structure, spectral content, and underlying neuronal activity. Thus, interpreting the information measures obtained with LZC should take into account the specific algorithm used to coarse-grain the signal. Further theoretical work is needed to determine whether k-means is a superior coarse-graining method than the median in terms of capturing the dynamics of the original signal in the symbolization process.

One of the main results of this study was the finding that early sleep characterized by high values of SWA is characterized by reduced LZC of the LFP signal. The mechanisms underlying the homeostatic increase of SWA are unclear, but may depend on the synaptic strength within local and global cortical networks (Tononi and Cirelli 2014; Vyazovskiy et al. 2011a), along with other factors, such as the levels of arousal-promoting neuromodulation (Polack et al. 2013; Steriade 1993; Steriade et al. 1993). Our results suggest an intriguing possibility that the two factors can be disentangled by focusing on the information content rather than on the absolute values of EEG SWA. Indeed, we found that there was no simple one-to-one correspondence between the values of LFP SWA or neuronal firing rates and the information content in the corresponding LFP signal or spike trains. Notably, even after the epochs were matched by SWA, the values of LZC were still substantially lower during the initial sleep after sleep deprivation. This is an intriguing observation that may provide an important tool to distinguish “unspecific” changes in SWA, which may result from changes in the level of arousal-promoting neuromodulators, from functional changes in synaptic connectivity.

A novel unexpected finding was that the average levels of LZC derived from waking LFP were stable across the 4 h of sleep deprivation, while epoch-to-epoch variability in LZC values increased progressively. It is possible that this dissociation reflects an overall increase in cortical activity in the course of SD, which is necessary to maintain the level of arousal typical for awake state, or increased neuronal excitability, which are reflected in high LZC, while frequent intrusions of sleep-like activity typical for a sleep-deprived brain, result in an occurrence of individual epochs characterized by low LZC. Future studies are necessary to address whether increased state instability revealed with LZC is associated with predictable behavioral or cognitive deficits. Recently, useful insights were obtained by looking at network activity during and after sleep deprivation using critical neural dynamics. Specifically, it was found that sustained waking was associated with a disarrangement of cascading dynamics of neuronal avalanches, and these “fading” signatures of criticality have been restored by sleep (Meisel et al. 2013). It was suggested that this reflects renormalization of optimal computational capabilities necessary for information processing and learning, which are impaired after sleep deprivation and restored after sleep.

To our knowledge, this is the first study where LZC was used to characterize the dynamics of sleep SWA, which is not only one of the essential defining characteristics of physiological NREM sleep, but is also a valuable marker of sleep depth, preceding sleep-wake history, and local and global synaptic/spiking activity (Vyazovskiy and Delogu 2014). It is well known, however, that absolute values of SWA (or delta-power) are determined by a number of factors [for a review, see Davis et al. (2011)]. For example, in the early developmental age, SWA is overall substantially higher than compared with adults, and the increase in SWA after sleep deprivation is “blunted” (Kurth et al. 2010; Nelson et al. 2013). The mechanisms and implications of this observation are yet unclear (de Vivo et al. 2014), and the progress has been hindered by the overall changes in the frequency content and the amplitude of the signals in young children and adults, which are difficult to interpret. Conversely, the most striking change in sleep with aging is the reduction in the number and amplitude of sleep slow waves (Carrier et al. 2011; Feinberg and Campbell 2003), K-complexes, and sleep spindles (Colrain et al. 2011; Crowley et al. 2002). Therefore, it is suggested that slow-wave sleep, as scored according to conventional criteria, is essentially absent in the elderly. Moreover, substantial changes in the amount of slow-wave sleep and/or the amplitude of SWA were reported between sexes and ethnic groups (Carrier et al. 2001; Durrence and Lichstein 2006; Latta et al. 2005; Mokhlesi et al. 2012), which may be indicative of differences in sleep quality. Even between individuals within a relatively homogenous group, interindividual variability in spectral EEG amplitude is often pronounced, which often necessitates using a normalization procedure. LZC may appear a useful approach to avoid the procedure of normalization, as it is on one hand independent of absolute amplitude, and, as we showed in the present study, shows much lower interindividual variability. Therefore, metrics such as LZC may be more informative and capture functional changes in the signal that are otherwise hidden by substantial changes in signal amplitude.

An interesting novel observation was that, while, on average, a negative association was found between multiunit activity and LZC, the relationship with the activity of specific well-isolated neurons often deviated substantially from the rest of the population. We can only speculate about specific neuronal subtypes in this case, but it is tempting to suggest that the narrow spike-width of those neurons, which overall correlated positively with LZC, is indicative of inhibitory neurons, which are essential for network dynamics associated with increased information content in waking and REM sleep. Thus, investigating metrics, sensitive to spatio-temporal microstructure of network dynamics, may appear crucial for understanding the functional significance of changes in brain activity between vigilance states, within a state, or in pathology (Feige et al. 2013; Meisel et al. 2013; Strelets et al. 2003; Vyazovskiy et al. 2014).

An important implication of our results is that LZC may provide information beyond what can be obtained with power spectral analysis, which corroborates earlier studies. For example, a recent study, in which two nonlinear indexes of complexity—LZC and approximate entropy—were used, showed that these metrics were more sensitive in detecting drug-induced changes in brain activity in patients with brain injury compared with spectral power (Valenza et al. 2011). Notably, LZC appears also to be more sensitive than some other nonlinear metrics. Specifically, in another study, LZC of the EEG was used to estimate the depth of anesthesia in patients under sevoflurane, isoflurane, propofol, or desflurane, in which responsiveness was estimated behaviorally with the Observer's Assessment of Alertness/Sedation (OAA/S) score (Zhang et al. 2001), and it was found that LZC outperformed approximate entropy, spectral entropy, and median frequency, reaching accuracies greater than 90% in predicting behavior from brain activity. Another valuable insight has been provided in the context of Alzheimer's disease. Specifically, it has been shown that EEG analysis with LZC can differentiate patients with Alzheimer's disease from control subjects with accuracy >80% (Abásolo et al. 2006). Furthermore, LZC has also been proven useful in the prediction of epileptic seizures (Radhakrishnan and Gangadhar 1998) and in the characterization of abnormal changes in mental arithmetic tasks in schizophrenia and depression (Li et al. 2008).

Our study has several methodological and conceptual limitations that must be addressed in future studies. First, the recordings have been obtained in one rodent species only, and both LFP and neuronal data were obtained from deep layers of one cortical area. Therefore, caution is warranted in generalizing these findings to other brain regions, anatomically distinct from the cortex, and other species, especially humans, in which the EEG is usually recorded from the scalp, and the underlying neuronal activity data are not available. Second, it should be kept in mind that the signals under scrutiny usually represent just a tiny fraction of the infinitely complex brain dynamics occurring at many spatio-temporal scales and produced by multiple independent and interacting components (Olbrich et al. 2011; Vyazovskiy and Delogu 2014), which poses a significant challenge for interpreting their origin and functional significance. Third, although our results suggest that LZC may appear extremely useful and provide important insights into the mechanisms underlying large-scale changes in EEG signals, at present, it remains a speculation.

In summary, computing LZC values for the LFP across spontaneous sleep and waking, and during and after sleep deprivation, revealed systematic changes of potential interest. We conclude that the LZC of the local field potential and neuronal spike trains can provide unique insights into the network mechanisms underlying the response of the brain to sleep loss, and pave the way toward gaining better understanding of their physiological and functional relevance.

## GRANTS

This work was supported by National Institute of Mental Health Grant 1R01MH099231 (to G. Tononi), the Engineering and Physical Sciences Research Council Grant EP/I000992/1 (to D. Abásolo and V. V. Vyazovskiy), and Wellcome Trust Strategic Award 098461/Z/12/Z (Sleep and Circadian Neuroscience Institute).

## DISCLOSURES

No conflicts of interest, financial or otherwise, are declared by the authors.

## AUTHOR CONTRIBUTIONS

Author contributions: D.A., G.T., and V.V.V. conception and design of research; D.A., S.S., R.M.d.S., and V.V.V. analyzed data; D.A., G.T., and V.V.V. interpreted results of experiments; D.A., S.S., and V.V.V. drafted manuscript; D.A., G.T., and V.V.V. edited and revised manuscript; D.A., S.S., R.M.d.S., G.T., and V.V.V. approved final version of manuscript; V.V.V. performed experiments; V.V.V. prepared figures.
